# CXCL12 is involved in α-synuclein-triggered neuroinflammation of Parkinson’s disease

**DOI:** 10.1186/s12974-019-1646-6

**Published:** 2019-12-12

**Authors:** Yuanyuan Li, Mengyue Niu, Aonan Zhao, Wenyan Kang, Zhichun Chen, Ningdi Luo, Liche Zhou, Xiongwei Zhu, Liming Lu, Jun Liu

**Affiliations:** 1 Department of Neurology & Institute of Neurology, Ruijin Hospital Affiliated to Shanghai Jiaotong University School of Medicine, Shanghai, 200025 China; 2 Department of Pathology, Case Western Reserve University, 2103 Cornell Road, Cleveland, OH USA; 3 Shanghai Institute of Immunology, Shanghai Jiaotong University School of Medicine, Shanghai, 200025 China; 4 CAS Center for Excellence in Brain Science and Intelligence Technology, Ruijin Hospital Affiliated to Shanghai Jiaotong University School of Medicine, Shanghai, 200025 China

**Keywords:** α-Synuclein, CXCL12, Microglia, Parkinson’s disease

## Abstract

**Background:**

The mechanisms underlying the pathogenesis and progression of Parkinson’s disease (PD) remain elusive, but recent opinions and perspectives have focused on whether the inflammation process induced by microglia contributes to α-synuclein-mediated toxicity. Migration of microglia to the substantia nigra (SN) could precede neurodegeneration in *A53T* mice. We hypothesized that CXCL12 could be a mediator in the α-synuclein-induced migration of microglia.

**Methods:**

After establishing appropriate animal and cell culture models, we explored the relationship between α-synuclein and CXCL12 in *A53T* mice, primary microglia, and BV-2 cell lines. We also explored the mechanisms of these interactions and the signaling processes involved in neuroinflammation.

**Results:**

We confirmed the positive correlation between α-synuclein and CXCL12 in the postmortem brain tissue of PD patients and the upregulated CXCR4 expression in SN microglia of *A53T* mice. In addition, as expected, α-synuclein increased the production of CXCL12 in microglia via TLR4/IκB-α/NF-κB signaling. Importantly, CXCL12/CXCR4/FAK/Src/Rac1 signaling was shown to be involved in α-synuclein-induced microglial accumulation.

**Conclusions:**

Our study suggests that CXCL12 could be a novel target for the prevention of α-synuclein-triggered ongoing microglial responses. Blocking CXCL12/CXCR4 may be a potential therapeutic approach for PD progression.

## Background

Parkinson’s disease (PD) is one of the most common neurodegenerative disorders, affecting 2~3% of the population aged 65 years and older [1]. Histological degeneration of dopaminergic neurons in the substantia nigra (SN) and the formation of Lewy bodies (LBs) containing aggregates of α-synuclein are the main neuropathological hallmarks. The underlying pathogenetic mechanism of PD remains elusive, but recent opinions and perspectives on the subject have focused on the inflammation process induced by microglia in the SN, which contributes to α-synuclein-mediated toxicity [2–4]. One hypothesis is that the course of PD may spiral out of control with excessive microglial activation and overproduction of cytokines and other inflammatory mediators [5].

Evidence for the ongoing activation of microglia in PD has been obtained using positron emission tomography (PET) imaging with ^11^C-PK11195 labeling of active microglia [6]. Widespread microglial activation with increased binding of ^11^C-PK11195 has been reported in the pons, basal ganglia, and frontal and temporal cortices in PD patients compared to those in normal controls [7]. In addition, studies have demonstrated a positive correlation between the levels of ^11^C-PK11195 in the midbrain and the Unified Parkinson’s Disease Rating Scale (UPDRS) score in early-stage PD patients [8, 9]. Moreover, microglia have been found to preferentially colocalize with aggregated α-synuclein in the SN in PD animal models [10–13]. These findings suggest that excessive migration of microglia in the SN may contribute to the progression of PD. However, the mechanism by which this ongoing process is involved in the progression of PD remains unknown.

α-Synuclein is produced in presynaptic regions of neurons, and its toxicity is associated with dysfunction of multiple pathways; moreover, α-synuclein can be secreted into the extracellular space and activate microglia to release proinflammatory and chemoattractant cytokines, inducing dopaminergic neurodegeneration [14–19]. Previous studies have mainly focused on its direct triggering of microglia; however, α-synuclein can also recruit microglia to the SN and induce constant activation by affecting microglial motility, thus inadvertently damaging neurons [16, 20, 21]. Although a recent study suggested that neuronal α-synuclein could potentially induce microglial migration in a CD11b-dependent manner [22], the explosive and cascading amplification of aggregated microglia in the SN and the potential mechanisms involved have not been explained.

In our current study, we found that CXCL12 was upregulated in SN tissue from postmortem PD patients and *A53T* (α-synuclein mutant) mice. α-Synuclein could promote the secretion of CXCL12 by microglia via the TLR4/IκB-α/NF-κB pathway. Furthermore, CXCL12 was involved in α-synuclein-induced microglial migration through binding to CXCR4. Finally, we confirmed that FAK/Src mediated CXCL12-triggered microglial directional migration by upregulating GTP-bound Rac1 activation, resulting in microglial migration towards the SN and continuous microglial activation.

## Methods

### Human brain samples

Formalin-fixed and paraffin-embedded postmortem human brain sections were obtained from the Human Brain and Spinal Fluid Resource Center (HBSFRC) at the VA West Los Angeles Healthcare Center. Both PD (*n* = 5) and control (*n* = 5) brain samples were from age-matched (between 60 and 90 years of age) males and females. All PD cases were clinically diagnosed as sporadic PD with a similar severity and without other known neurological diseases. Control subjects had no history of neurological illness or brain trauma.

### Reagents

Medium and supplements for cell culture were purchased from Gibco (Grand Island, USA). Purified human recombinant α-synuclein was purchased from rPeptide (Athens, GA, USA). The peptide was dissolved in sterile ddH_2_O (Grand Island ile) to create a 1 mg/ml (75 μM) stock solution. Dissolved in water, rH α-syn (endotoxin level, < 0.024 EU/μg) was incubated with agitation at 37 °C for 7 days, allowing the formation of oligomers; this technique was used in previous studies [22, 23]. Recombinant murine CXCL12 was purchased from PeproTech (Rocky Hill, USA). TRIzol Reagent was purchased from Life Technologies, and PrimeScript RT Master Mix and SYBR® Premix Ex Taq^TM^ qPCR SuperMix were purchased from Takara. A Dynabeads Antibody Coupling Kit was obtained from Life Technologies (Grand Island, USA). Magnetic-activated cell sorting (MACS) isolation kits were purchased from Miltenyi Biotech (Germany). Transwell inserts were obtained from Corning-Costar (Tewksbury, MA, USA).

### Cell culture and treatment

The immortalized murine microglial cell line BV-2 and murine RAW 264.7 cell line was maintained at 37 °C in a 5% CO_2_ humidified incubator in DMEM supplemented with 10% FBS and 100 U/ml PS. For most experiments, cell suspensions were seeded into six-well plates (10^6^ cells/ml) and treated with 250 nM α-synuclein (prepared as described above) or 100 ng/ml recombinant murine CXCL12 protein for the indicated times. AMD3100 (1 ng/ml, TargetMol, USA) was used as a CXCR4 antagonist, TAK242 (Merck, Germany) was used as a TLR4 inhibitor at a concentration of 100 nM, pyrrolidine dithiocarbamate (PDTC, 50 μM, TargetMol) was applied as an inhibitor of NF-κb (nuclear factor kappa), and C29 was used as a blocker of TLR2 signaling (50 μM, TargetMol). The cells were harvested for further analysis after stimulation for 12, 24, or 48 h, and the cell supernatants (500 μl) were collected for further analysis of CXCL12 levels by ELISA.

Primary microglia from neonatal mice were magnetically separated using MACS technology (Miltenyi Biotec, USA) according to the manufacturer’s instructions. Briefly, brain tissues of 12 neonatal mice were obtained under sterile conditions. Single-cell suspensions were prepared from the tissues and incubated with magnetic beads conjugated with anti-CD11b antibody (BioLegend, USA). Then, the cell suspensions were applied to the MACS column; the magnetically labeled CD11b+ cells were attracted to the magnet in the mass spectrometry (MS) column, while the unlabeled cells were eluted. Isolated microglia were plated onto 24-well plates at a density of 1 × 10^5^ cells per well and cultured in DMEM/F12 medium (Life Technologies, USA) supplemented with 10% FBS and 100 U/ml PS. After 24 h, the culture medium was replaced with fresh medium for 2 to 4 days, and the cells were then treated with 250 nM α-synuclein for 24 h. The cell supernatants were collected for further analysis of CXCL12 levels by ELISA.

### Animals

Transgenic mice (B6;C3­Tg(Prnp­SNCA*A53T)83Vle/J) expressing human A53T mutant *α-synuclein* were originally obtained from the Jackson Laboratory (J004479) and maintained at the Experimental Animal Center at Shanghai Jiaotong University School of Medicine in accordance with specific pathogen-free (SPF) standards. Offspring were intercrossed to generate homozygous transgenic mice and *WT* mice. All animals were housed in their home cages and maintained on a reverse day/night cycle by artificially manipulating the light in the room. Brain tissues were collected from the SNs of 3-month-old mice, and primary microglia were sorted from 12 neonatal mice per experiment. All animal experiments were performed in accordance with the Guidelines of the Laboratory Animal Ethical Standards of Shanghai Jiaotong University School of Medicine.

### Quantitative reverse transcription qPCR

Brain tissue, BV-2 cells, or primary microglia were homogenized in TRIzol Reagent, and total RNA was then extracted from the homogenate according to the manufacturer’s protocol. The mRNA was reverse transcribed into cDNA, and 2 μl of cDNA was subjected to real-time qPCR assays with SYBR® Premix Ex Taq^TM^ qPCR SuperMix. The two-step amplification protocol consisted of denaturation for 30 s at 95 °C followed by 40 cycles of 95 °C for 5 s and 60 °C for 60 s. The target mRNA levels were quantified by real-time qPCR on a ViiA 7 System. The relative levels of the mRNAs were quantified and normalized to those of GAPDH. The primer sequences are as follows:

qPCR primers for CXCL12: F: 5′-TGCATCAGTGACGGTAAACCA-3′; R: 5′-TTCTTCAGCCGTGCAACAATC-3′

qPCR primers for CXCR4: F: 5′-GAAGTGGGGTCTGGAGACTAT-3′; R: 5′-TTGCCGACTATGCCAGTCAAG-3′

qPCR primers for TLR1: F: 5′-TGAGGGTCCTGATAATGTCCTAC-3′; R: 5′-TGAGGGTCCTGATAATGTCCTAC-3′

qPCR primers for TLR2: F: 5′-GCAAACGCTGTTCTGCTCAG-3′; R: 5′-GCAAACGCTGTTCTGCTCAG-3′

qPCR primers for TLR4: F: 5′-AGGCGTCTCCCTCTATTGTATT-3′; R: 5′-GAGGCCAATTTTGTCTCCACA-3′

qPCR primers for GAPDH: F: 5′-AGGTCGGTGTGAACGGATTTG-3′; R: 5′-TGTAGACCATGTAGTTGAGGTCA-3′

### Western blotting

Western blotting was performed as described previously [24]. Brain tissues, BV-2 cells, primary microglia, and RAW264.7 cells were lysed in RIPA lysis buffer containing protease and phosphatase inhibitor cocktails and 1 mM phenylmethanesulfonyl fluoride (PMSF, Beyotime, China) for 30 min on ice. Total cell extracts were centrifuged at 14,000*g* for 15 min at 4 °C and assayed with a BCA protein assay kit. For western blot analysis, 20 μg of lysate per lane was separated by 8–12% polyacrylamide gel electrophoresis and transferred to PVDF membranes. Next, the membranes were incubated overnight at 4 °C with the following primary antibodies: anti-CXCR4 (Abcam, 1:1000, USA), anti-TLR4 (Proteintech, 1:1000, USA), anti-pIκb-α/Iκb-α (Cell Signaling Technology, 1:1000, USA), anti-histone 3 (Cell Signaling Technology, 1:1000, USA), and anti-NF-κB p65 (nuclear/cytoplasmic) (Abcam, 1:1000, USA). Immunoreactive bands were visualized using secondary antibodies conjugated to HRP (Santa Cruz, CA, 1:10000, USA). Anti-β-actin (Sigma-Aldrich, 1:5000, USA) was used as a control. Band density was visualized using chemiluminescence, and images were captured with a Tanon 5200 Multi chemiluminescent imaging system (Tanon, Shanghai, China). Signal intensity was quantified with ImageJ (National Institutes of Health).

### Immunostaining

Mice were perfused intraventricularly with PBS followed by 4% PFA. The brains were post-fixed in PFA overnight at 4 °C, cryoprotected for 2 successive days in 20% and 30% sucrose, and gradually frozen at − 20 °C. Coronal sections (30 μm thick) were cut using a cryostat (Leica CM1850) and stored at − 20 °C. A schematic showing the positions of the brain immunostaining pictures was given in Additional file 1: Figure S1a. The contours of the SN were delimited using a low-magnification (× 10) objective and defined on the basis of TH immunostaining (Additional file 1: Figure S1b). Labeling was performed on three sections at the level of the SN for each mouse. Sections (4 μm) of formalin-fixed, paraffin-embedded brain samples from the SN were prepared and stained by applying a conventional avidin-biotin complex technique. Primary antibodies were used at dilutions of 1:800 and 1:400 to detect CXCL12 and CXCR4, respectively, and HRP-conjugated secondary antibodies were applied to amplify the signal. DAB substrate or AEC substrate was added for final staining. Images were acquired with an inverted microscope. For immunofluorescence assessment, sections were incubated with primary antibodies targeting IBA-1 (Abcam, USA), CXCR4, and actin. Secondary antibodies conjugated with Alexa Fluor 488, 594, and 647 were used to mark the targets. Images were acquired with a confocal laser scanning microscope (Carl Zeiss, Oberkochen, Germany).

### Flow cytometry

Flow cytometry (FCM) was used to quantitate the frequency of microglia (CD11b+). To create cell suspensions of the SN brain area of *A53T* and *WT* mice (*n* = 3), tissues were cut into small pieces and mechanically dissociated in PBS. Then, the cell suspensions were filtered through a 40-mm cell strainer, washed three times, and stained with the following monoclonal antibodies: anti-CD11b FITC and anti-CXCR4 APC (BioLegend, USA). To verify the sorting efficiency of primary microglia, single-cell suspensions before and after MACS were prepared as previously described. The cells were then stained with anti-CD11b FITC and subjected to FCM analysis using FlowJo software. FCM analysis was performed using a Canto-DIVA, and the data were subsequently analyzed using FlowJo software version 7.6.1 (TreeStar, Ashland, OR, USA).

### Transwell migration assay

The effects of α-synuclein and CXCL12 on microglia migration were assessed using polycarbonate membrane inserts with 8.0 μm pores (Corning, USA) placed in a 24-well plate as previously described [25]. Images of the migrated cells on the lower surface of the insert were captured under a phase-contrast microscope. The number of transmigrated cells was determined using an inverted microscope at × 200 magnification with five fields of view using ImageJ software. The experiment was repeated three times.

### ELISA

The conditioned medium (500 μl) collected from BV-2 cells or primary microglia was evaluated for the production of CXCL12 using ELISA (BD Biosciences, San Jose, USA) according to the manufacturer’s instructions. The optical density of each well was determined using a microplate reader at 405 nm, and the amount of CXCL12 was calculated from the standard curve.

### Statistical analysis

Statistical comparisons between two groups were performed with Student’s *t* tests. For analysis of more than two groups, one-way ANOVA was performed using GraphPad Prism 7.0 software. The data are shown as the mean ± SEM for three independent experiments; for the animal experiments, *n* = 5 for most data points. *p* < 0.05 was considered to indicate statistical significance.

## Results

### CXCL12/CXCR4 was overexpressed in the brain tissues of PD patients and *A53T* transgenic mice

**Fig. 1:**
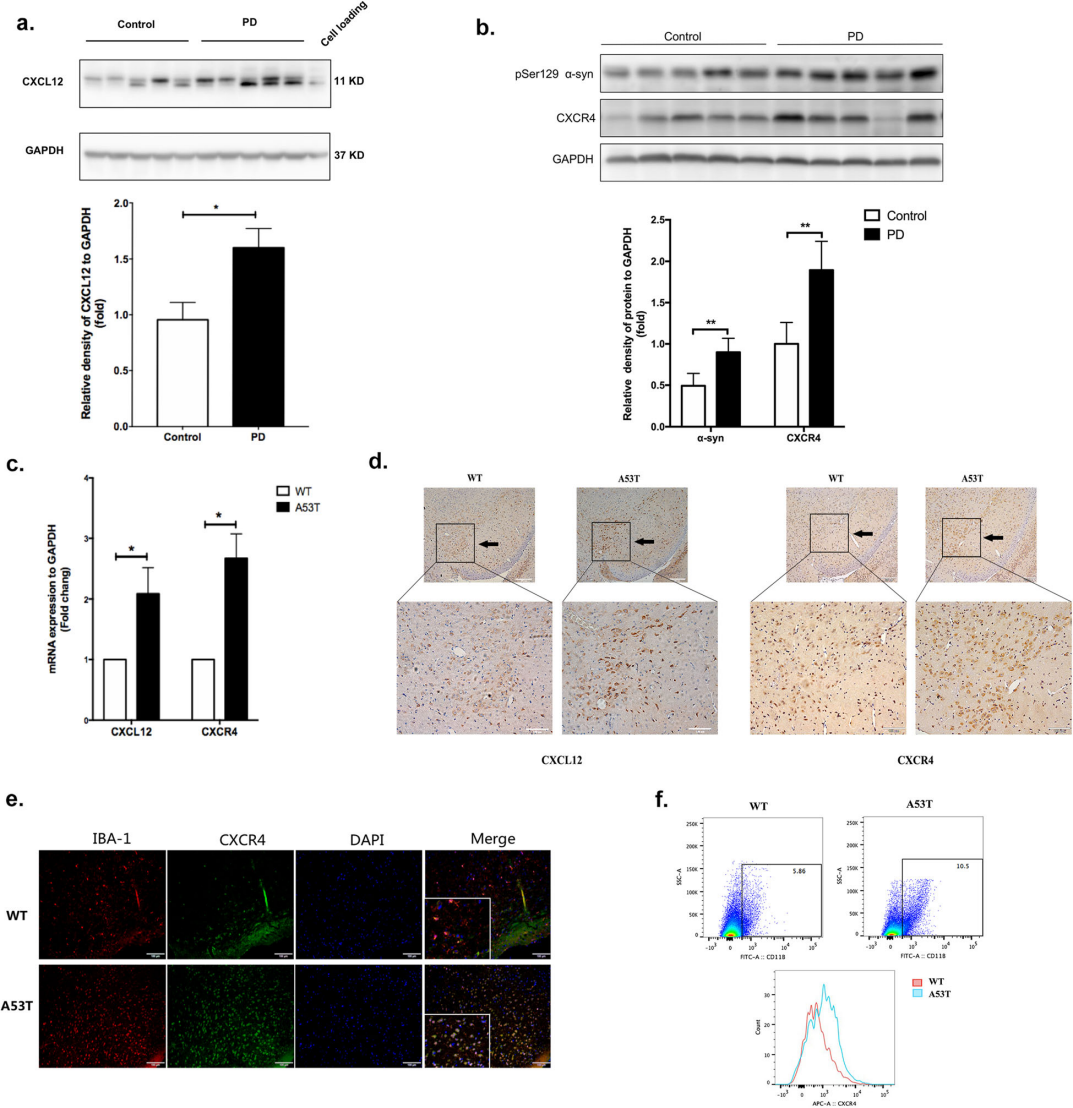
The levels of CXCL12 and CXCR4 were elevated in the brain tissues of PD patients and *A53T* transgenic mice. **a** Western blot analysis showed the expression of CXCL12 in SN sections of postmortem brain tissue from control and PD subjects. GAPDH was used as an internal control for gel loading, and M17 cell protein was loaded as a positive control. **b** Western blot analysis showed an upregulation of CXCR4 and pser129 a-syn protein levels in human PD brains compared with the control. **c** The mRNA expression of CXCL12 and CXCR4 was determined by real-time qPCR in A53T and WT mice. *n* = 3 per group. **d** Brain slices from the substantia nigra were stained for CXCL12 and CXCR4 by immunohistochemistry. *n* = 3 per group for *A53T* and *WT* mice. **e** Brain slices from the substantia nigra were double stained for IBA-1 (red) and CXCR4 (green). Images were captured with a fluorescence microscope. Scale bar = 100 μm. *n* = 3 per group. **f** Brain tissue from the substantia nigra was stained for microglia (CD11b-FITC) and CXCR4 (APC), and the frequency of the CD11b+ and CD11b+CXCR4+ cells was assessed by flow cytometry. *n* = 3 per group for *A53T* and *WT* mice. The data are shown as the mean ± SEM for three independent experiments. **p < 0.01. *p < 0.05

As indicated by a previous study, CXCL12 and CXCR4 are upregulated in both the postmortem brain tissues of PD patients and in MPTP-treated mice [26]. Thus, we confirmed the expression of CXCL12 in SN sections from postmortem brain tissue of patients with PD and healthy controls. Unlike the healthy controls, all of the PD patients showed positive expression of LBs (α-synuclein) (Table 1). Firstly, we found CXCL12 expression was significantly enhanced in PD patients, as expected (*p* = 0.01, Fig. 1a). Secondly, we verified the expression of pSer129 α-synuclein and the receptor of CXCL12, CXCR4 in control and PD groups. Similarly, PD patients exhibited higher expression of pSer129 α-synuclein in the SN section, with CXCR4 elevated as well (*p <* 0.01, Fig. 1b). Furthermore, we verified the role of CXCL12 and CXCR4, in α-synuclein transgenic (A53T) mice, revealing that compared to those in *WT* mice, the mRNAs of both CXCL12 and CXCR4 were overexpressed in the SN brain tissue section in *A53T* mice (*p* < 0.05, Fig. 1c). In addition, IHC showed that the expression of CXCL12 and CXCR4 was enhanced in brain tissue sections from *A53T* mice (Fig. 1d; Additional file 1: Figure S1), confirming the positive correlation between α-synuclein and CXCL12/CXCR4. Microglia were originally identified as targets of CXCL12 based on the binding of CXCR4. Thus, we further performed immunofluorescence staining to study the expression of CXCR4 in microglia, revealing colocalization between IBA-1 and CXCR4 in the SN of *A53T* mice (Fig. 1e). Simultaneously, we performed FCM to determine the frequency of CXCR4-expressing microglia. As shown, the frequency of CXCR4 positivity (CD11b+CXCR4+) appeared to be generally elevated in microglial subpopulations from *A53T* mice (*p* = 0.04, Fig. 1f), consistent with the increased proportion of CD11b+ microglia in *A53T* mice compared to that in *WT* mice.

**Table 1:** Demographic characteristics of the subjects enrolled in the autopsy

	Group	Age	Gender	Lewy body	Substantia nigra
1	Control	61	Male	No	Yes
2	Control	79	Male	No	Yes
3	Control	67	Male	No	Yes
4	Control	90	Male	No	Yes
5	Control	83	Female	No	Yes
6	PD	77	Female	Yes	Yes
7	PD	87	Male	Yes	Yes
8	PD	74	Female	Yes	Yes
9	PD	87	Female	Yes	Yes
10	PD	81	Female	Yes	Yes

### α-Synuclein upregulated the expression of CXCL12 in microglia

**Fig. 2:**
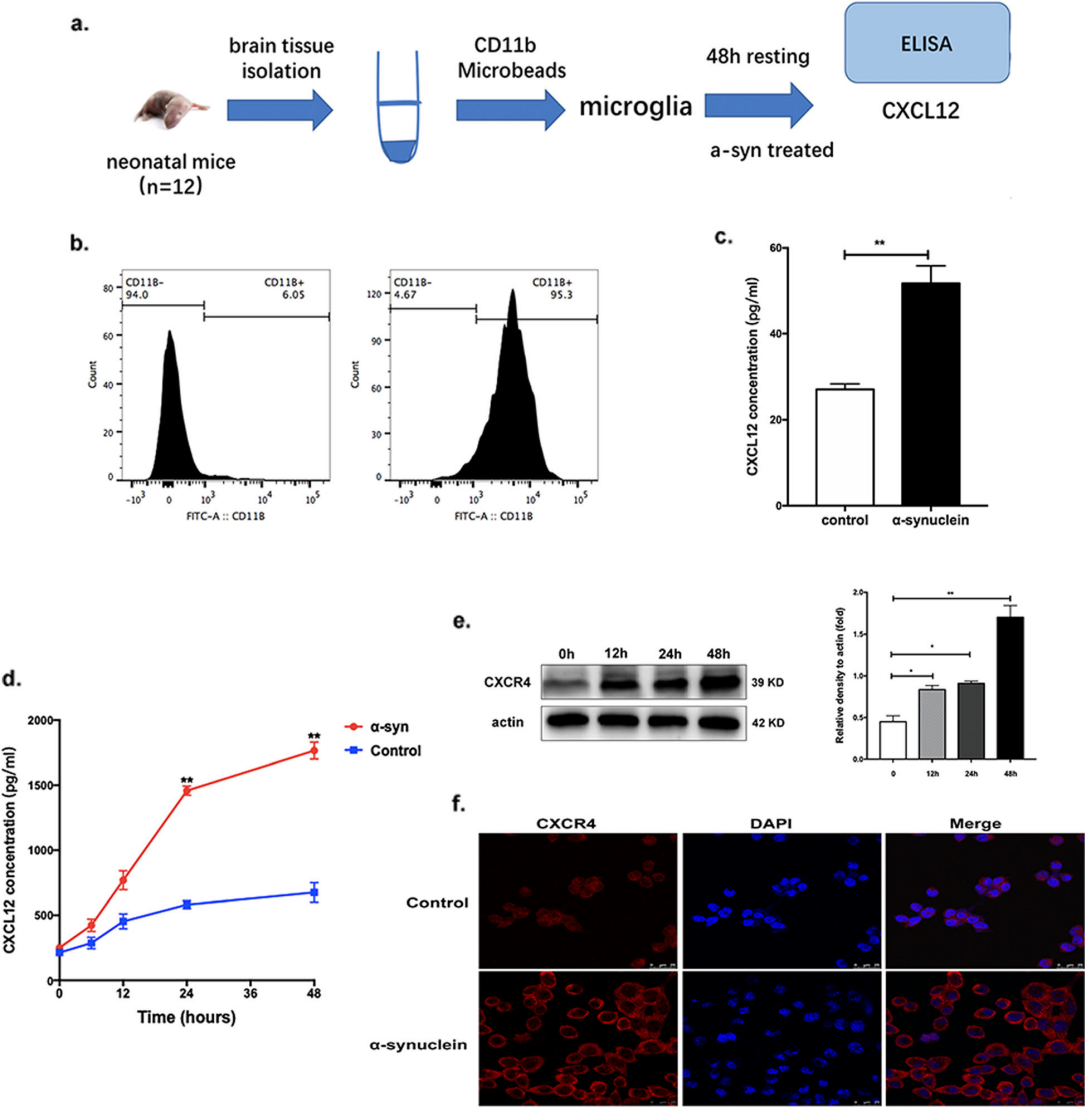
α-Synuclein upregulated the expression of CXCL12 in microglia. **a** Primary microglia were sorted by anti-CD11b-conjugated microbeads from 12 neonatal mice once. **b** The sorting efficiency was assessed by flow cytometry. **c** The concentration of CXCL12 in the supernatant of primary microglia was measured by ELISA after stimulation by α-synuclein or BSA. **d** BV-2 cells were stimulated by α-synuclein or BSA; supernatants were collected at 6, 12, 24, and 48 h; and the concentration of CXCL12 was evaluated by ELISA. **e** The expression of CXCR4 in BV-2 cells was determined by western blot after stimulation by α-synuclein. **f** BV-2 cells were stained for CXCR4 (red) after stimulation for 48 h with α-synuclein. Images were captured with a fluorescence microscope. Scale bar = 25 μm. The data are shown as the mean ± SEM for three independent experiments. **p < 0.01. *p < 0.05. BSA served as a control

To verify whether the upregulated CXCL12 originated from microglia, we sorted neonatal mouse primary microglia using anti-CD11b-conjugated microbeads (Fig. 2a). After sorting, the proportion of CD11b+ cells was increased to 95.3% with a high efficiency (Fig. 2b). Firstly, we found that the levels of TNF alpha, IL-1b, and Il-6 were all increased in α-synuclein-treated groups, which indicated an active phenotype of primary microglia (Additional file 1: Figure S2). As expected, after stimulation by α-synuclein, the production of CXCL12 in the supernatant of the stimulated group was markedly upregulated compared with that in the supernatant of the control group (*p* < 0.01, Fig. 2c), which suggested that α-synuclein could promote the secretion of CXCL12 by microglia. Furthermore, BV-2 cells were also treated with α-synuclein, and CXCL12 was measured in the supernatant by ELISA. As shown in Fig. 2d, α-synuclein significantly increased the production of CXCL12 at 24 and 48 h post-treatment (*p = 0.*003 and *p* = 0.008, respectively). The expression of CXCR4 was also detected by western blot and was found to be markedly upregulated in stimulated BV-2 cells compared to that in control cells after 12, 24, and 48 h of stimulation, especially after 48 h (*p* = 0.02, *p* = 0.01, and *p* = 0.005 for 12, 24, and 48 h, respectively, Fig. 2e and Additional file 1: Figure S3a), consistent with the CXCR4 immunofluorescence results (*p* < 0.01, Fig. 2f and Additional file 1: Figure S3b).

### α-Synuclein induced the secretion of CXCL12 via TLR4/IκB-α/NF-κB signaling

**Fig. 3:**
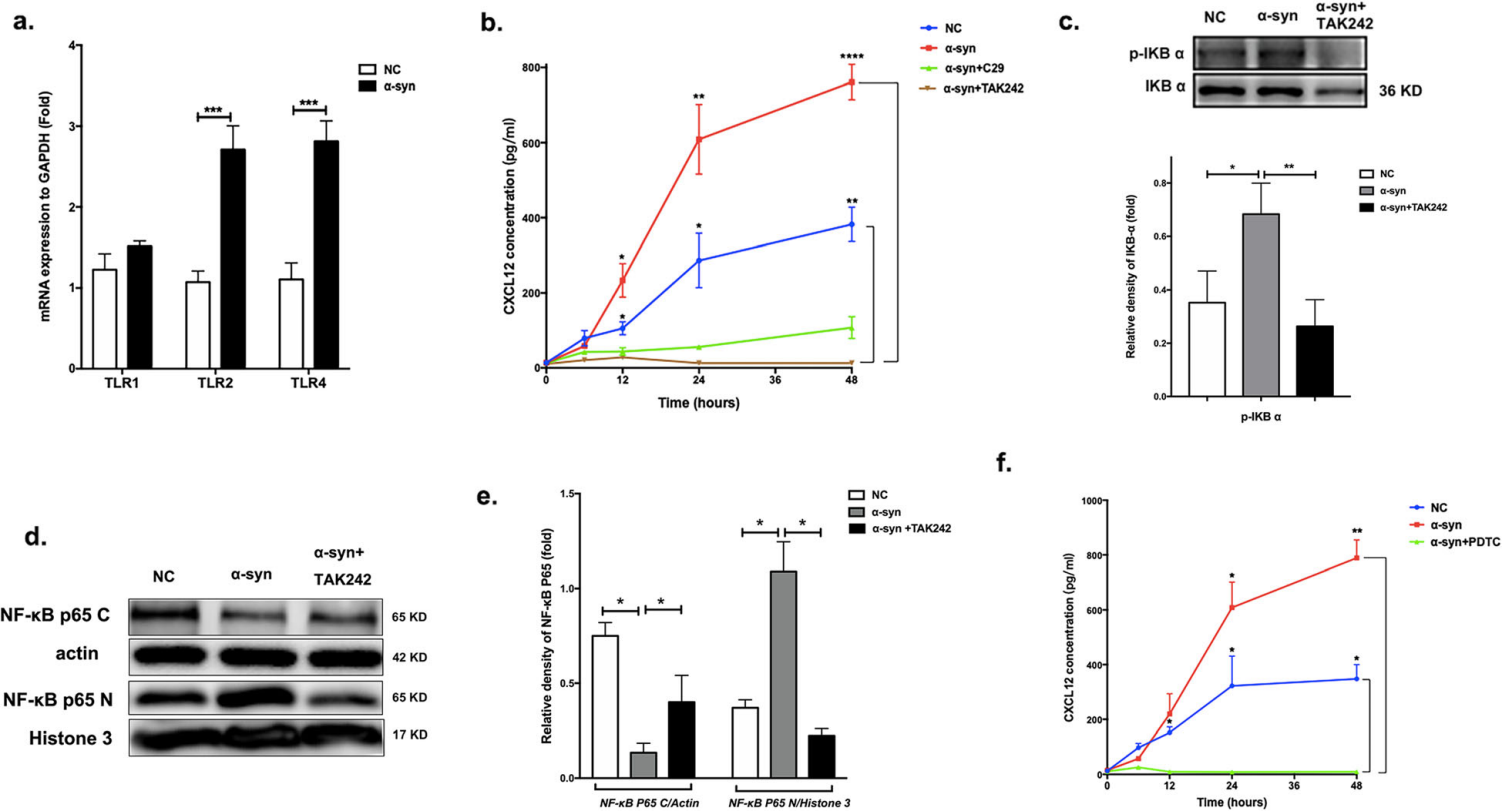
α-Synuclein could induce the microglial secretion of CXCL12 via TLR4/IκB-α/NF-κB signaling. **a** Real-time qPCR was used to analyze the mRNA expression levels of TLR1, TLR2, and TLR4. **b** BV-2 cells were preincubated with TAK242 or C29 for 2 h, and the CXCL12 levels in supernatants (500 μl) collected after 12, 24, 36, or 48 h of stimulation with α-synuclein were evaluated by ELISA. **c** The phosphorylation of IκB-α in BV-2 cells was detected by western blot after 24 h of incubation with α-synuclein with or without TAK242. **d**, **e** The levels of NF-κB p65 complex in the nucleus (NF-κB p65 N) and cytoplasm (NF-κB p65 C) were detected by western blot after 24 h of stimulation with α-synuclein with or without TAK242. Actin served as the control for NF-κB p65 C, while histone 3 served as the control for NF-κB p65 N. **f** BV-2 cells were preincubated with PDTC for 2 h, and the CXCL12 levels in supernatants collected after 12, 24, 36, and 48 h of stimulation with α-synuclein were evaluated by ELISA. The data are shown as the mean ± SEM from three independent experiments. ****p < 0.0001. ***p < 0.001. **p < 0.01. *p < 0.05. BSA served as a control

Furthermore, we investigated the detailed mechanism of CXCL12 secretion induction by α-synuclein. As previously suggested, toll-like receptor (TLR) signaling is a major pathway mediating inflammation in microglia and has increasingly been implicated in PD [27–29]. Specifically, recent studies have shown that TLR4 is upregulated in microglia and that α-synuclein can activate proinflammatory TLR4 pathways [30]. Therefore, we hypothesized that α-synuclein could activate TLRs mediating the production of CXCL12. To test this hypothesis, microglia were treated with α-synuclein for 24 h, and the mRNA expression levels of TLR1/2/4 were analyzed. The expression of both TLR2 (*p* < 0.001) and TLR4 (*p* < 0.001) was enhanced, while that of TLR1 was not consistently altered (Fig. 3a). We further examined whether TLR2 or TLR4 was involved in CXCL12 production by adding specific inhibitors during conditioning with α-synuclein. Treatment with the specific TLR4 inhibitor TAK242 markedly reduced CXCL12 levels in the supernatant compared to that in supernatants treated with α-synuclein alone or with a negative control, while treatment with the TLR2 inhibitor C29 caused no obvious change (Fig. 3b); these results suggested that TLR4 was involved in the expression of CXCL12.

Previous studies have indicated that TLR4 can induce the phosphorylation and degradation of IκB-α (inhibitor of NF-κB) and promote the translocation of the transcription factor complex NF-κB to the nucleus [30], followed by cytokine expression. To explore whether this pathway is involved in the production of CXCL12, we examined the effect of α-synuclein on IκB-α phosphorylation and NF-κB p65 translocation. Indeed, after 24 h of incubation with α-synuclein of BV-2 cells, the expression of phosphorylated IκB-α (pIκB-α) was increased, while it was changed in the opposite direction after treatment with TAK242 (*p* < 0.05, Fig. 3c), an inhibitor of TLR4. As previously reported, NF-κB can translocate to the nucleus after IκB-α activation and mediate inflammatory responses [31, 32]. In this study, the cytoplasmic levels of the NF-κB p65 complex were decreased and the nuclear levels were enhanced after 24 h of α-synuclein treatment (*p* < 0.05). In addition, a change in the opposite direction was observed when TLR4 was inhibited by TAK242 (*p* < 0.05; Fig. 3d, e). Finally, we measured CXCL12 levels after inhibiting NF-κB activation with the specific inhibitor PDTC, and a marked reduction in CXCL12 was observed (Fig. 3f). The above results suggested that α-synuclein induces CXCL12 production by activating the TLR4/NF-κB pathway through phosphorylation of IκB-α. Similarly, the increased expression of IκB-α and nuclear levels NF-κB p65 was verified in primary microglia, during the production of CXCL12 stimulated by α-synuclein (Additional file 1: Figure S4**)**. The control condition of p-IKB and NF-KB p65 expression stimulated by TAK242 were shown in Additional file 1: Figure S5a and Figure S5b, with no changes effected, which indicated that those effects above were indeed mediated by the exogenously added α-synuclein. While for CXCL12 levels, there seems to be a trend of inhibition on CXCL12 comparing to control in BV-2 cells, which may be the inhibition of cell proliferation induced by the inhibitors themselves (Additional file 1: Figure S5c).

### CXCL12/CXCR4 participated in α-synuclein-induced microglial migration

**Fig. 4:**
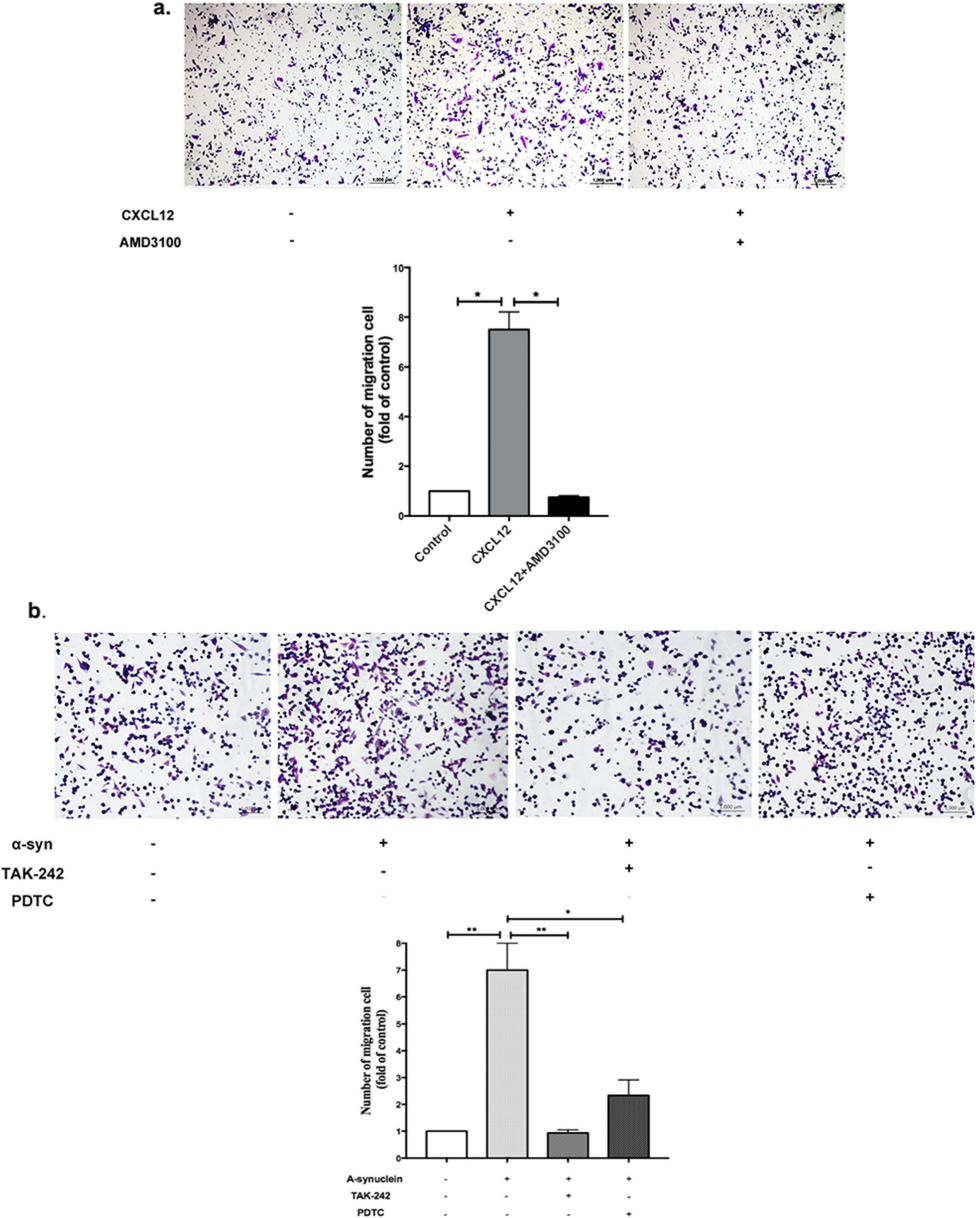
CXCL12/CXCR4 was involved in α-synuclein-induced microglial migration. **a** The migration of BV-2 cells towards CXCL12 was measured by Transwell assay, and AMD3100 was added to the upper chambers to block the interaction between CXCL12 and CXCR4. **b** The migration of BV-2 cells induced by α-synuclein was observed with or without pretreatment with a TLR4 inhibitor (TAK242) or an NF-κB inhibitor (PDTC). The data are shown as the mean ± SEM from three independent experiments. *p < 0.05

Previous studies have indicated that α-synuclein might direct microglial migration [22]. CXCL12/CXCR4 ligand/receptor binding is involved in many physiological processes, mainly chemotaxis. Our results suggested that α-synuclein-induced microglial migration is mediated by CXCL12/CXCR4 signals. We first observed the migration of microglia induced by α-synuclein and CXCL12. As shown, CXCL12 could significantly induce the migration of BV-2 cells (*p* < 0.05); however, this effect was decreased when AMD3100, a well-characterized and specific antagonist for CXCR4, was added to the upper chamber (*p* < 0.05, Fig. 4a). In addition, migration induced by α-synuclein was eliminated by pretreatment with a TLR4 inhibitor (TAK242) or NF-κB inhibitor (PDTC) (Fig. 4b), which further suggested that α-synuclein induced microglial migration by promoting the production of CXCL12 via the TLR4/IκB-α/NF-κB pathway.

### CXCL12/CXCR4 mediated the migration of microglia through FAK/Src/Rac-1 signaling

**Fig. 5:**
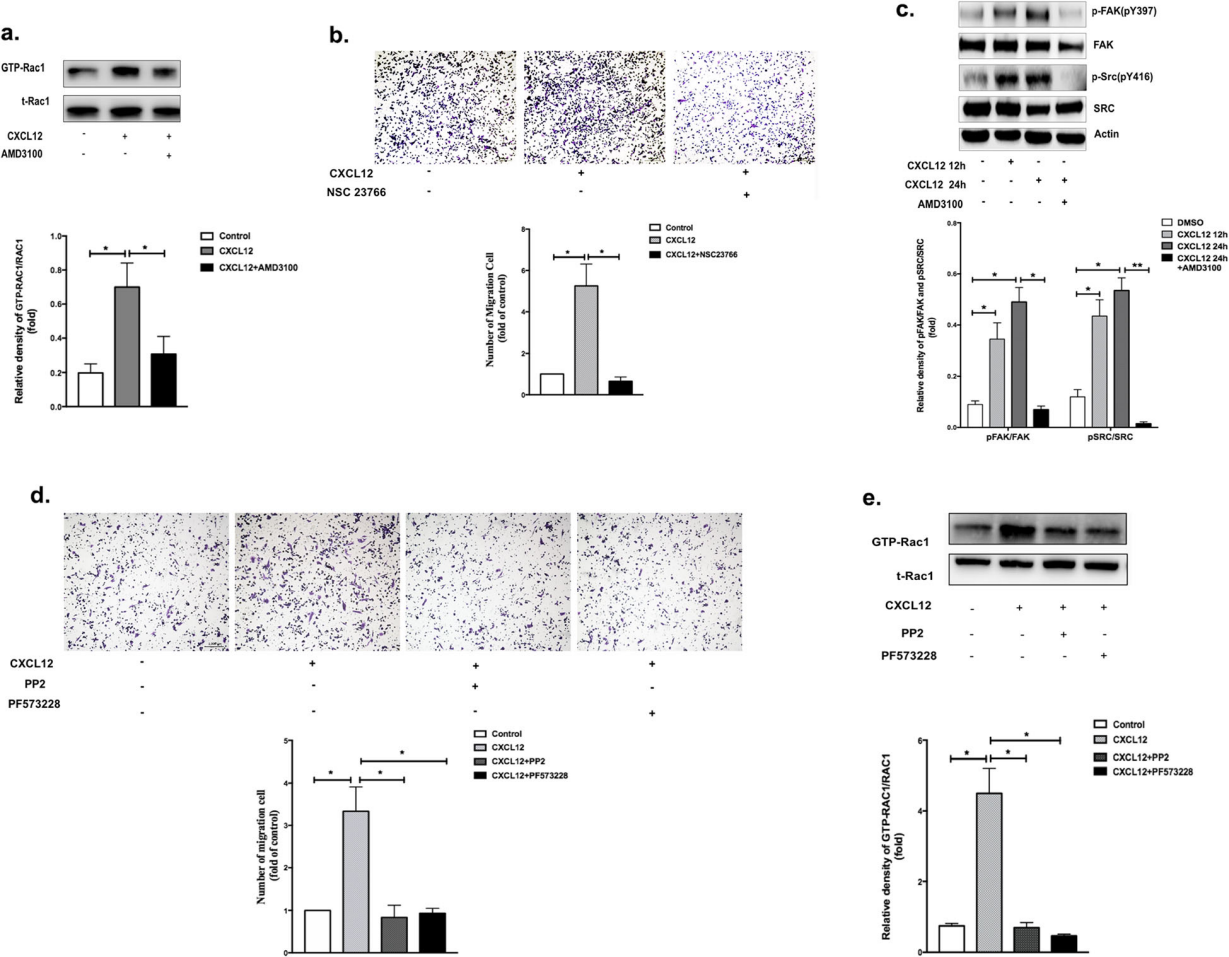
CXCL12/CXCR4 mediated the migration of microglia through FAK/Src/Rac-1 signaling. **a** The expression levels of GTP-Rac1 and total Rac1 were detected by western blotting after 24 h of stimulation by CXCL12 with or without AMD3100. **b** BV-2 cells were pretreated with NSC23766 for 2 h, and the migration of BV-2 cells towards CXCL12 was measured by the Transwell assay. **c** Western blot analysis was used to assess the expression levels of phospho-FAK (pY397), FAK, phospho-Src (pY416), and Src after 24 h of stimulation with CXCL12. **d** Migration of primary microglia towards CXCL12 with or without PP2 or PF573228 was measured by the Transwell assay. **e** Expression of GTP-Rac1 and total Rac1 were detected by western blotting after 24 h of stimulation by CXCL12 with or without PP2 or PF573228. The data are shown as the mean ± SEM from three independent experiments. ****p < 0.0001. ***p < 0.001. **p < 0.01. *p < 0.05. BSA served as a control


Since CXCL12 plays an important role in the migration of microglia induced by α-synuclein, we observed the dynamic migration of BV-2 microglial cells incubated with CXCL12 (Additional file 2: Video S1). However, the mechanism of migration is unknown. Because Rac1 is an important signaling molecule that could promote the protrusion of lamellipodia followed by cell migration [33, 34], we further examined whether Rac1 activation occurred in response to CXCL12/CXCR4, both in BV-2 cells and primary microglia. The active form of Rac1 was measured by precipitating active GTP-bound Rac1 with a GST-PAK-binding domain (GST-PBD). As shown in Fig. 5a and Additional file 1: Figure S6a, binding to CXCL12 stimulated a marked increase in Rac1 activity, which declined after the addition of AMD3100. In addition, treatment with NSC23766 (an inhibitor of Rac1) significantly reduced the CXCL12-induced microglia migration compared to that in the control (Fig. 5b).

**Additional file 2: Video S1.** Migration of BV-2 cells towards CXCL12.


Previous studies have shown that the signaling pathways of focal adhesion kinase (FAK) and Src are crucial for remodeling the cytoskeleton and mediating cell mobility by activating Rho GTPases [35–37]. Thus, to ascertain the involvement of FAK/Src signaling pathways in CXCL12-promoted microglial migration, the phosphorylated statuses of FAK and Src in BV-2 cells were firstly examined. As expected, significant increases in phospho-FAK (pY397) and phospho-Src (pY416) were observed in BV-2 cells exposed to CXCL12 for 12 or 24 h compared to those in control cells, but these increases were blocked by AMD3100 (Fig. 5c and Additional file 1: Figure S6b). To confirm that FAK/Src is responsible for CXCL12-triggered migration, BV-2 cells were preincubated with the FAK-specific inhibitor PF573228 or the Src inhibitor PP2 for 2 h. As shown in Fig. 5d and Additional file 1: Figure S6c, the CXCL12-induced migration of BV-2 cells and primary microglia was diminished by both inhibitors. Additionally, to determine whether Rac1 is the downstream target of FAK/Src, we tested GTP-Rac1 activation in response to CXCL12 pretreated with PF573228 and PP2. The results showed a significant decrease in the activation of GTP-Rac1 in the pretreated BV-2 cells (Fig. 5e), which indicated that GTP-Rac1-induced microglial migration was regulated by FAK/Src. The above experiments were performed in primary microglia and the similar trends were also confirmed for GTP-Rac1 (Additional file 1: Figure S6d).

Furthermore, we verified the FAK/Src/Rac-1 signaling induced by α-synuclein in primary microglia and RAW 264.7 cells. The RAW 264.7 cell is a macrophage cell line, which has been used with primary microglial cells to study the phenotypes of microglia treated by α-synuclein [38]. As was shown in Additional file 1: Figure S7 and Additional file 1: Figure S8, the expression of GTP-Rac1, p-FAK, and p-Src were all increased in α-synuclein stimulated group (CXCL12 overexpressed), while both AMD3100 and NSC23766 inhibited this effect. Taken together, these results confirmed that FAK/Src/Rac1 signaling was critical for CXCL12-induced microglial migration and indicated that the direct target of CXCR4 was upstream of this signaling pathway.

## Discussion

**Fig. 6:**
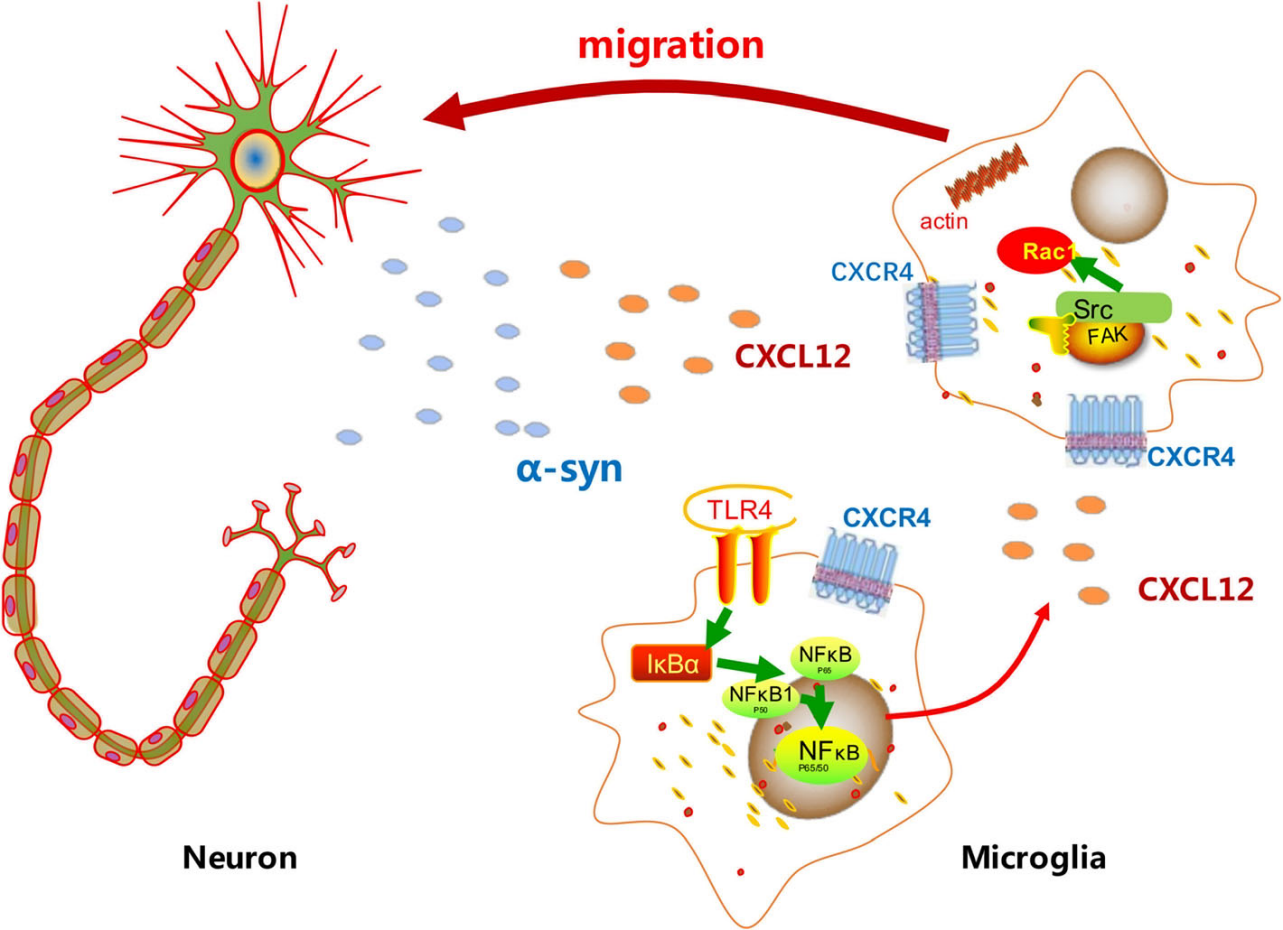
Schematic of the mechanism by which α-synuclein induces the accumulation of microglia through CXCL12/CXCR4. Released α-syn establishes a gradient in the space between the neurons and the microglia. α-Syn aggregates may bind to the TLR4 on microglia, eliciting an overexcretion of CXCL12 through TLR4/IκB-α/NF-κB signaling. CXCL12 participates in α-synuclein-induced microglial migration through binding to CXCR4 through the CXCL12/CXCR4/FAK/Src/Rac1 pathway. As a result, the microglia continue to migrate along the concentration gradient of α-syn and CXCL12 toward the sources of α-syn

Multiple studies have described the accumulation of reactive microglia in postmortem brain samples of PD patients [39–41]. As speculated, an array of activated microglia potentially coalesces in PD brains and drives disease progression via cytokine release [42]. It is well known that α-synuclein aggregates indirectly damage neurons by eliciting microglial-mediated neuroinflammation [43, 44]; however, the mechanism by which α-synuclein interacts with microglia and how the released cytokines play roles in this ongoing process remain incompletely understood. Our investigation has made several advances in elucidating how α-synuclein affects excessive microglial accumulation in the SN via cytokines. We found that CXCL12 was upregulated in microglia through TLR4/IκB-α/NF-κB signaling mediated by α-synuclein. CXCL12 participated in α-synuclein-induced microglial accumulation through the CXCL12/CXCR4/FAK/Src/Rac1 pathway (Fig. 6).

Studies focusing on the relationship between the α-synuclein peptide and the activation of microglia have gradually increased in recent years [23, 27–29, 45–47]. Neuroinflammation in *A53T* mice has been demonstrated; this state is marked by activated microglia and increased cytokine levels preceding neurodegeneration [48]. CXCL12 is a classic inflammatory chemokine and can be secreted by a variety of cells, including lymphocytes, monocytes, and endothelial cells, to bind to its receptor, CXCR4. CXCL12 and CXCR4 are constitutively expressed and widely detected in the CNS, especially in microglia, and have been shown to play important roles in the physiology of the brain and in pathological conditions, such as stroke, multiple sclerosis (MS), and some neurodegenerative diseases [49–51]. The relationship between CXCL12/CXCR4 and PD was first reported by Shimoji et al., who demonstrated that CXCL12/CXCR4 could participate in the etiology of PD [26]. Similarly, we herein found that CXCL12 was enhanced in the SNs of PD patients, consistent with α-synuclein aggregation (in LBs). This finding was further confirmed with *A53T* α-synuclein transgenic mice, in which both CXCL12 and CXCR4 were overexpressed in the SN. Furthermore, the frequency of CXCR4-positive cells was increased in microglial subpopulations of *A53T* mice. Collectively, these findings indicated a relationship among α-synuclein, CXCL12/CXCR4, and microglia.

CXCL12 has been observed in the cerebrospinal fluid (CSF) of patients suffering from an inflammatory neurological disorder [52, 53], and the levels of both CXCL12 and CXCR4 are upregulated in lipopolysaccharide (LPS)-pretreated microglia [54]. Thus, to clarify the role of CXCL12/CXCR4 in α-synuclein-related neuroinflammation, we first sorted primary microglia with anti-CD11b-conjugated microbeads and found elevated CXCL12 levels after α-synuclein pretreatment. Moreover, the expression of CXCR4 was enhanced along with strengthened secretion of CXCL2 in BV-2 cells. Given these findings, we propose that CXCL12/CXCR4 could mediate α-synuclein-triggered excessive microglial activation.

Furthermore, we explored the mechanism of CXCL12 upregulation in microglia. It has been proposed that microglial activation is induced by α-synuclein via binding to membrane receptors, such as integrin and TLR receptors [22]. TLR1, TLR2, and TLR4 are the major receptors expressed in microglia and have been indicated to mediate α-synuclein-dependent microglial activation processes, including phagocytic activity and proinflammatory cytokine release [46, 55, 56]. In the current study, we demonstrated that TLR4 plays a modulatory role in CXCL12 production triggered by α-synuclein. Inhibition of TLR4 markedly reduced the expression of CXCL12, which showed that the expression of CXCL12 induced by α-synuclein occurred via a TLR4-dependent pathway. These results are consistent with those of a previous study showing that downregulation of TLR4 inhibits resistin-inducing CXCL12 expression in human gastric cancer cell lines [57].

CXCL12/CXCR4 ligand/receptor binding is involved in many physiological processes, mainly chemotaxis. In our study, we also found that CXCL12 could induce the migration of BV-2 cells by binding to CXCR4. In addition, the migration induced by α-synuclein was eliminated by pretreatment with TAK242 or PDTC, which further indicated that α-synuclein induced microglial migration by promoting the production of CXCL12 via the TLR4/IκB-α/NF-κB pathway. Subsequently, using a Transwell coculture system, we explored the molecular events leading to microglial directional migration induced by CXCL12/CXCR4. As noted previously, FAK/Src is crucial for remodeling the cytoskeleton, while Rac-1 regulates multiple signaling pathways that control cytoskeletal organization, transcription, and cell proliferation. In this study, phospho-Src, phospho-FAK 397, and GTP-Rac1 were significantly upregulated upon CXCL12 treatment, and inhibitors of FAK, Src, and Rac could effectively diminish the migratory ability of microglia stimulated by CXCL12. These results suggest that CXCL12 triggers microglial migration via the FAK/Src/Rac1 axis.

In summary, we have elucidated that CXCL12/CXCR4 is involved in the interactions and signaling processes by which α-synuclein mediates the ongoing activation of microglia. Further studies are needed to explore the influence of the directional migration of microglia towards DA neurons induced by the α-synuclein/CXCL12/CXCR4 axis. With regard to AMD3100, an inhibitor of CXCR4 that has been approved by the FDA for autologous transplantation in patients with non-Hodgkin’s lymphoma (NHL) and multiple myeloma (MM) due to its effect on neutrophil mobilization [58], further studies are needed to explore its potential preventative effect on neuroinflammation in PD through inhibition of CXCR4.

## Conclusion

As illustrated in Fig. 6, we first suggest that CXCL12 could be a novel target for the prevention of α-synuclein-triggered ongoing microglial responses. CXCL12/CXCR4/FAK/Src/Rac1 signaling was shown to be involved in α-synuclein-induced microglial migration. However, further in vivo experiments are necessary to better comprehend the mechanistic effects of CXCL12/CXCR4 on the neuroinflammation in PD models. These findings suggest that blocking CXCL12/CXCR4 should be explored as a potential therapeutic strategy for PD patients.

## Supplementary information

**Additional file 1: Figure S1.** Location and standardized of SN position from mouse brain. **Figure S2.** Levels of inflammatory factors in α-synuclein and ADM3100 treated primary microglia. **Figure S3.** Expression of CXCR4 in BV-2 cells. (a) Western bolt showed no difference for different times of the expression of CXCR4 in controlled untreated BV-2 cells. (b) Quantitative fluorescence intensity of CXCR4 from various microscopy fields. **Figure S4.** Verification of TLR4/IκB-α/NF-κB signaling in primary microglia. (a) The phosphorylation of IκB-α was detected by western blot. (b) The levels of NF-κB p65 complex in the nucleus and cytoplasm were detected by western blot. (c) ELISA showed the CXCL12 levels in supernatants collected after stimulation with α- synuclein with or without inhibitors. **Figure S5.** Control condition of the inhibitors in BV-2 cells. (a)(b) Western bolt showed no difference of p-IKB and NF-KB p65 expression when TAK242 alone is added. (c) ELISA showed that CXCL12 expression was not affected by C29, TAK242 or PDTC alone. **Figure S6.** Verification of FAK/Src/Rac-1 signaling induced by CXCL12 in primary microglia. (a) The expression levels of GTP-Rac1 and total Rac1 were detected by western blotting. (b) Western blot showed the expression levels of phospho-FAK, FAK, phospho-Src and Src. (c) Migration of primary microglia towards CXCL12 with or without inhibitors was measured by the Transwell assay. (d) Expression of GTP-Rac1 and total Rac1 were detected by western blotting. **Figure S7.** Verification of FAK/Src/Rac-1 signaling induced by α-synuclein in primary microglia. (a)(b) Western blot analysis was used to assess the expression of phospho-FAK, FAK, phospho-Src, Src, GTP-Rac1 and total Rac1 after stimulation. **Figure S8.** Verification of FAK/Src/Rac-1 signaling induced by α-synuclein in RAW 264.7 cells. (a)(b) Western blot analysis was used to assess the expression of phospho-FAK, FAK, phospho-Src, Src, GTP-Rac1 and total Rac1 after stimulation.

## Data Availability

All data are provided in the manuscript and in the additional files.

## Abbreviations

CSF: Cerebrospinal fluid
FAK: Focal adhesion kinase
GST-PBD: GST-PAK-binding domain
IκB-α: Inhibitor of NF-κB
LBs: Lewy bodies
MACS: Magnetic-activated cell sorting
MM: Multiple myeloma
NF-κB: Nuclear factor kappa
NHL: Non-Hodgkin’s lymphoma
PD: Parkinson’s disease
PET: Positron emission tomography
PMSF: Phenylmethanesulfonyl fluoride
SN: Substantia nigra
SPF: Specific pathogen-free
TLR: Toll-like receptor
