# Supplementary material for: CXCL12 is involved in α-synuclein-triggered neuroinflammation of Parkinson’s disease
Source: J Neuroinflammation. 2019 Dec 12;16:263. doi: 10.1186/s12974-019-1646-6 (PMC6909602; doi:10.1186/s12974-019-1646-6)
Supplement: Supplementary file 2 — Additional file 1: Figure S1. Location and standardized of SN position from mouse brain. Figure S2. Levels of inflammatory factors in α-synuclein and ADM3100 treated primary microglia. Figure S3. Expression of CXCR4 in BV-2 cells. (a) Western bolt showed no difference for different times of the expression of CXCR4 in controlled untreated BV-2 cells. (b) Quantitative fluorescence intensity of CXCR4 from various microscopy fields. Figure S4. Verification of TLR4/IκB-α/NF-κB signaling in primary microglia. (a) The phosphorylation of IκB-α was detected by western blot. (b) The levels of NF-κB p65 complex in the nucleus and cytoplasm were detected by western blot. (c) ELISA showed the CXCL12 levels in supernatants collected after stimulation with α- synuclein with or without inhibitors. Figure S5. Control condition of the inhibitors in BV-2 cells. (a)(b) Western bolt showed no difference of p-IKB and NF-KB p65 expression when TAK242 alone is added. (c) ELISA showed that CXCL12 expression was not affected by C29, TAK242 or PDTC alone. Figure S6. Verification of FAK/Src/Rac-1 signaling induced by CXCL12 in primary microglia. (a) The expression levels of GTP-Rac1 and total Rac1 were detected by western blotting. (b) Western blot showed the expression levels of phospho-FAK, FAK, phospho-Src and Src. (c) Migration of primary microglia towards CXCL12 with or without inhibitors was measured by the Transwell assay. (d) Expression of GTP-Rac1 and total Rac1 were detected by western blotting. Figure S7. Verification of FAK/Src/Rac-1 signaling induced by α-synuclein in primary microglia. (a)(b) Western blot analysis was used to assess the expression of phospho-FAK, FAK, phospho-Src, Src, GTP-Rac1 and total Rac1 after stimulation. Figure S8. Verification of FAK/Src/Rac-1 signaling induced by α-synuclein in RAW 264.7 cells. (a)(b) Western blot analysis was used to assess the expression of phospho-FAK, FAK, phospho-Src, Src, GTP-Rac1 and total Rac1 after stimulation. [file 12974_2019_1646_MOESM1_ESM.docx]

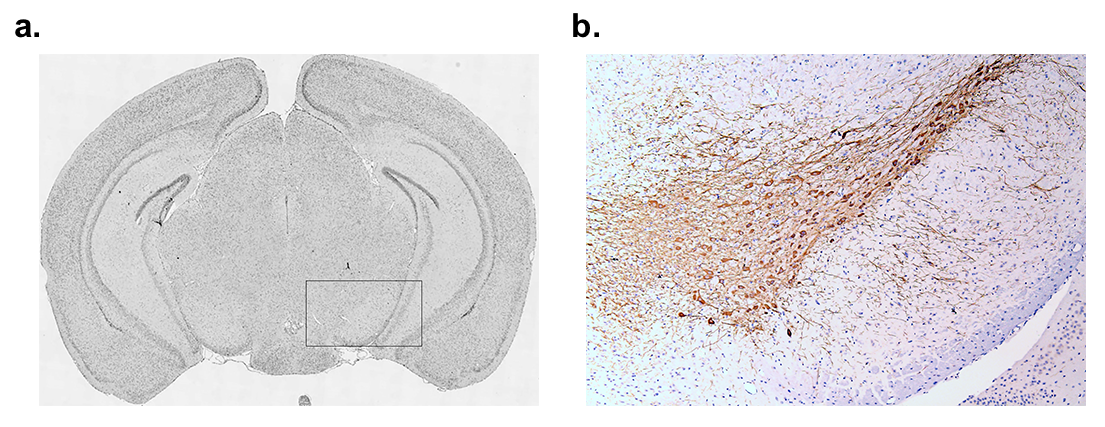


**Figure S1. Location and standardized of SN position from mouse brain** (a) Sketch picture showing the position of the immunostaining pictures obtained from brain. (b) TH positive immunostaining proved the SN position.

**
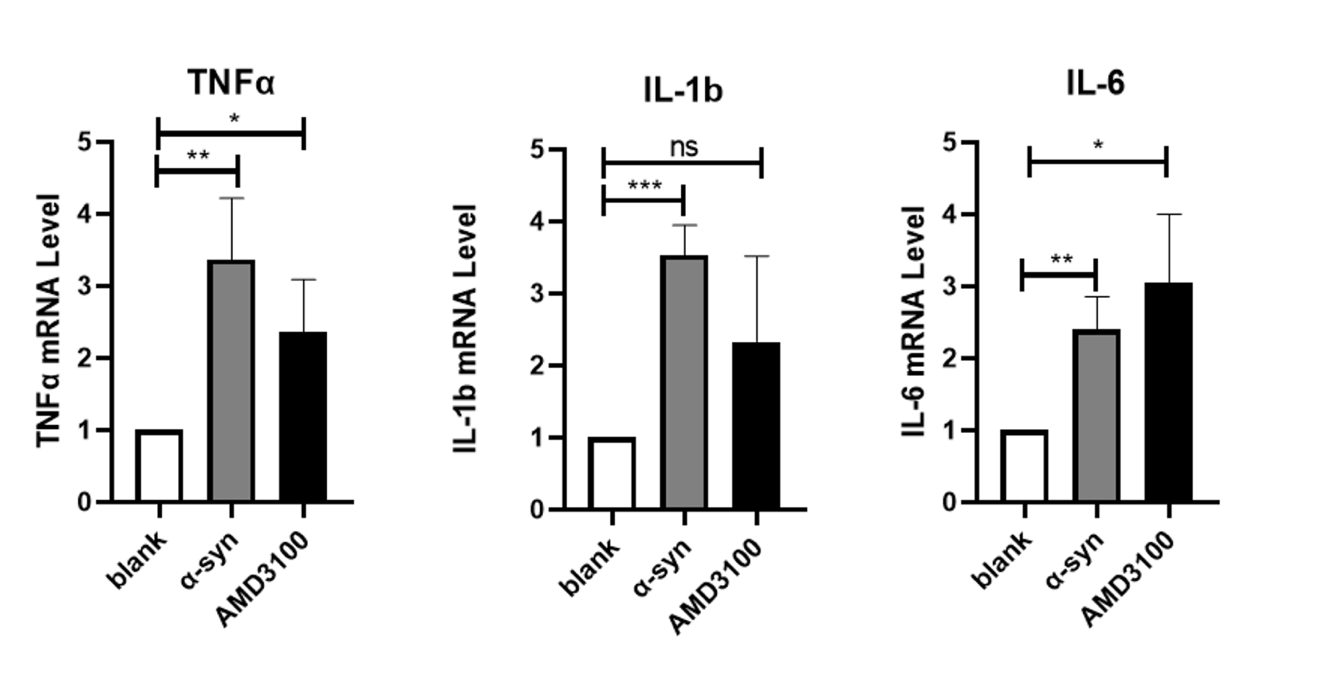
**

**Figure S2. Levels of inflammatory factors in α-synuclein and ADM3100 treated primary microglia**


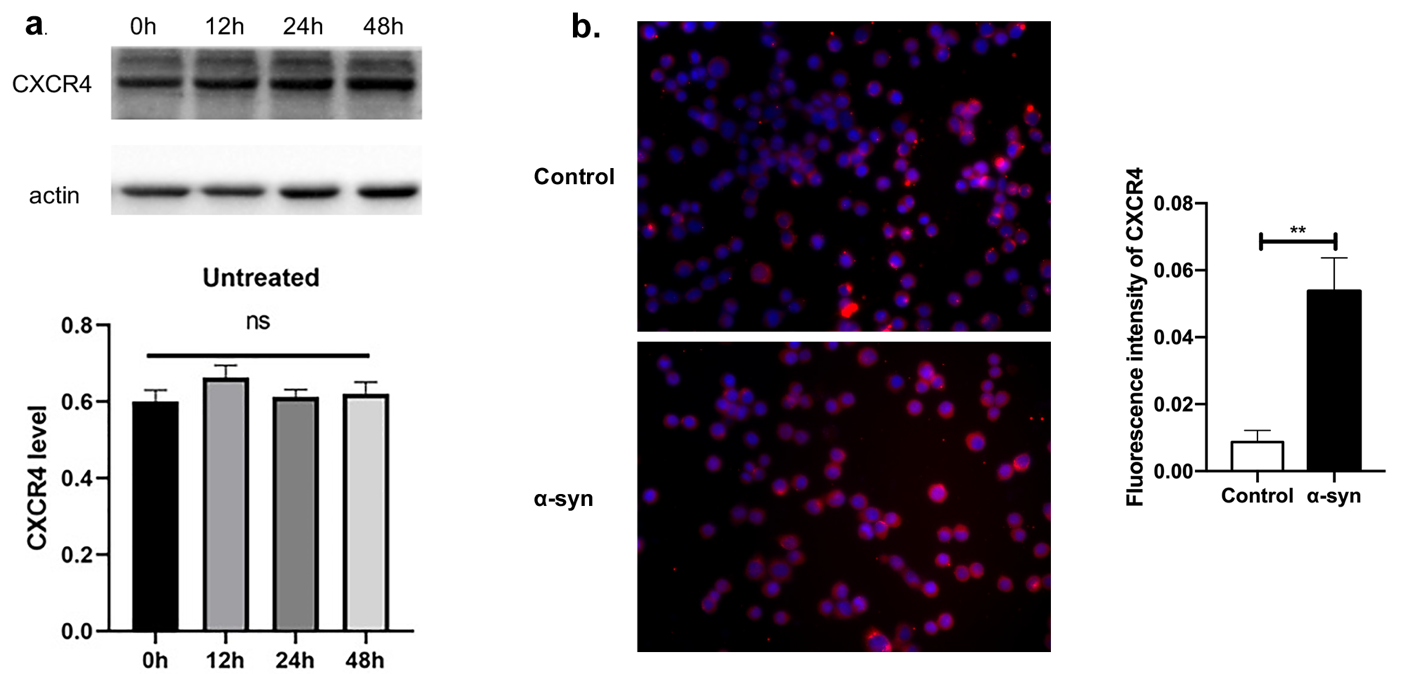


**Figure S3. Expression of CXCR4 in BV-2 cells.** (a) Western bolt showed no difference for different times of the expression of CXCR4 in controlled untreated BV-2 cells. (b) Quantitative fluorescence intensity of CXCR4 from various microscopy fields.

**
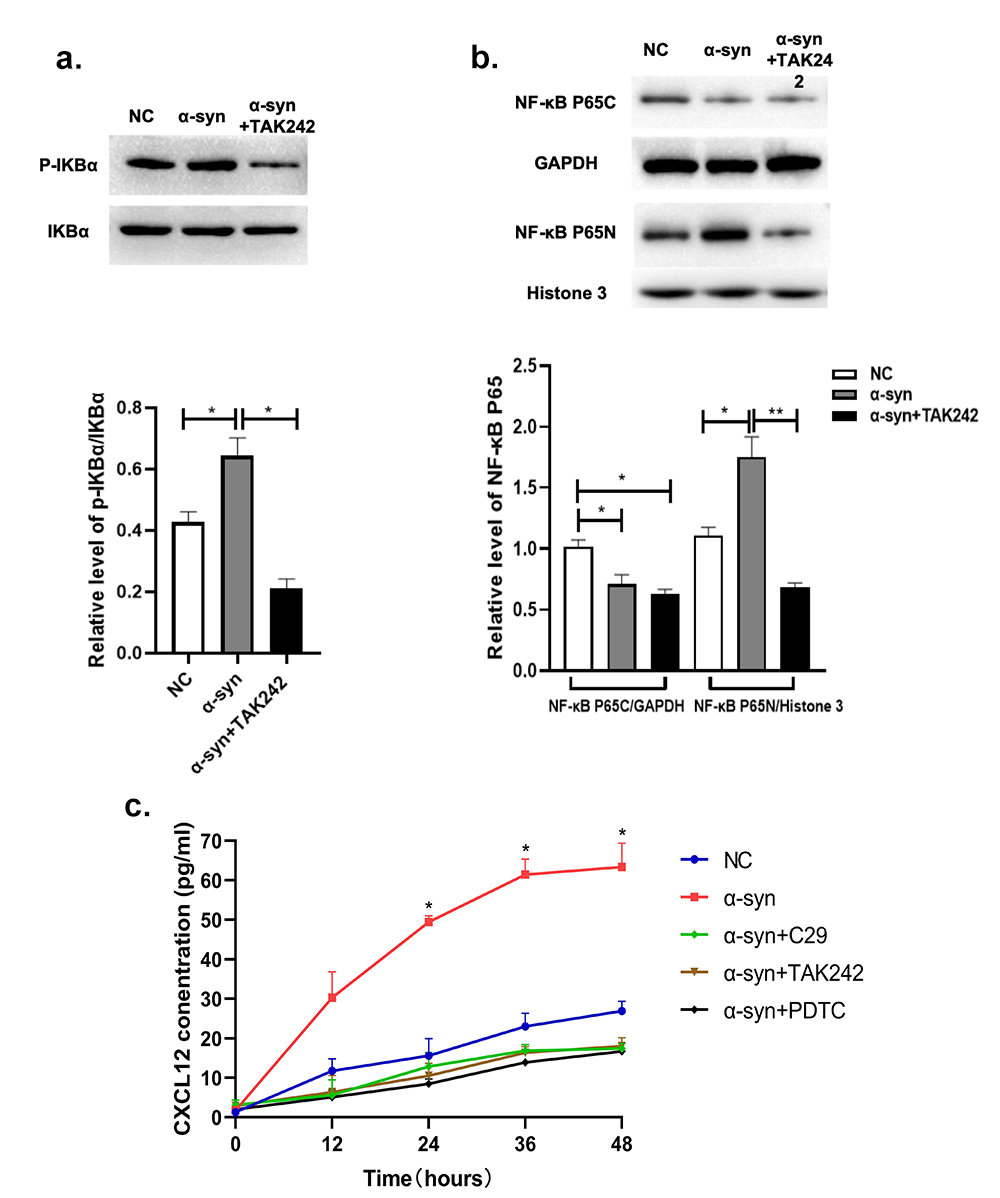
 Figure S4. Verification of TLR4/IκB-α/NF-κB signaling in primary microglia.** (a) The phosphorylation of IκB-α was detected by western blot after 24 h of incubation with α-synuclein with or without TAK242. (b) The levels of NF-κB p65 complex in the nucleus (NF-κB p65 N) and cytoplasm (NF-κB p65 C) were detected by western blot after 24 h of stimulation with α-synuclein with or without TAK242. Actin served as the control for NF-κB p65 C, while histone 3 served as the control for NF-κB p65 N. (c) ELISA showed the CXCL12 levels in supernatants collected after 12, 24, 36 and 48 h of stimulation with α-synuclein with or without inhibitors.

**
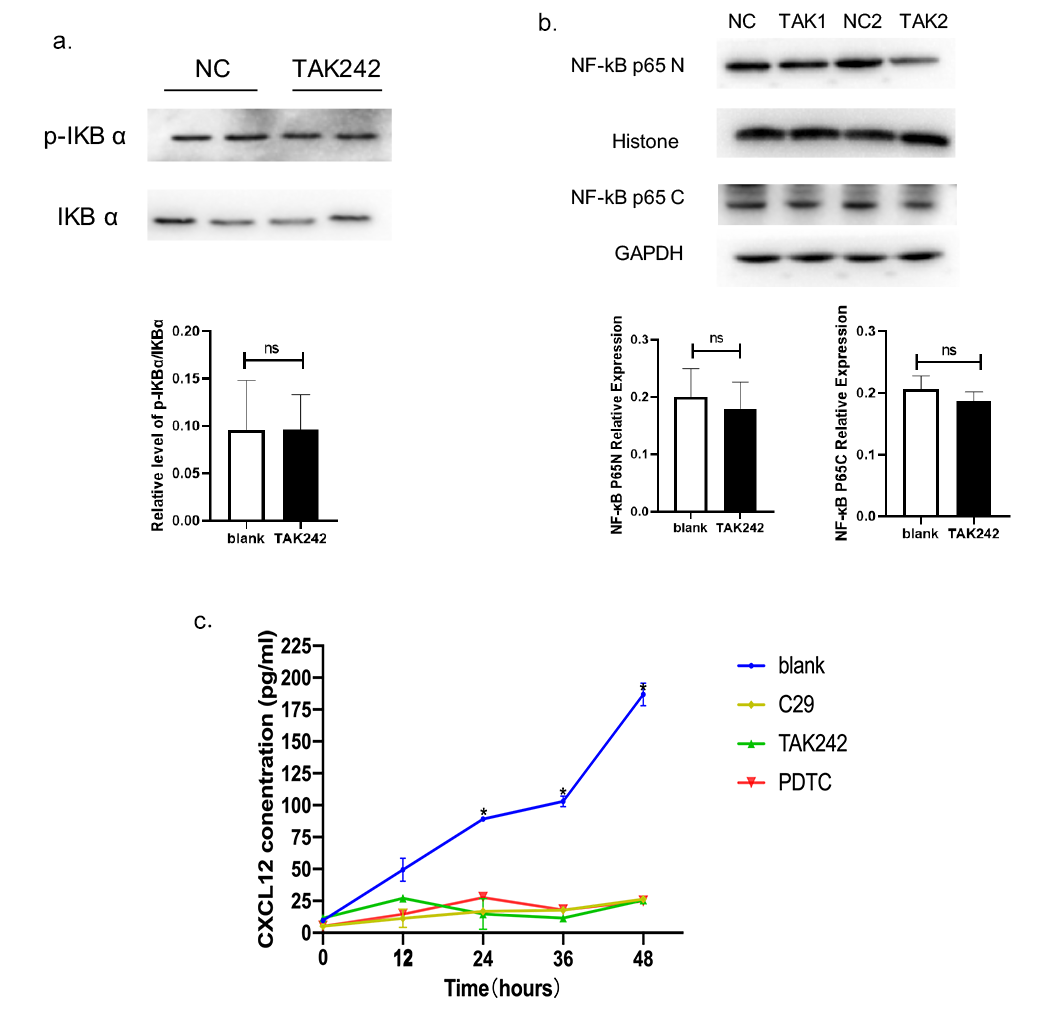
**

**Figure S5. Control condition of the inhibitors in BV-2 cells.** (a)(b) Western bolt showed no difference of p-IKB and NF-KB p65 expression when TAK242 alone was added. (c) ELISA showed that CXCL12 expression was inhibited by C29, TAK242 or PDTC.

**
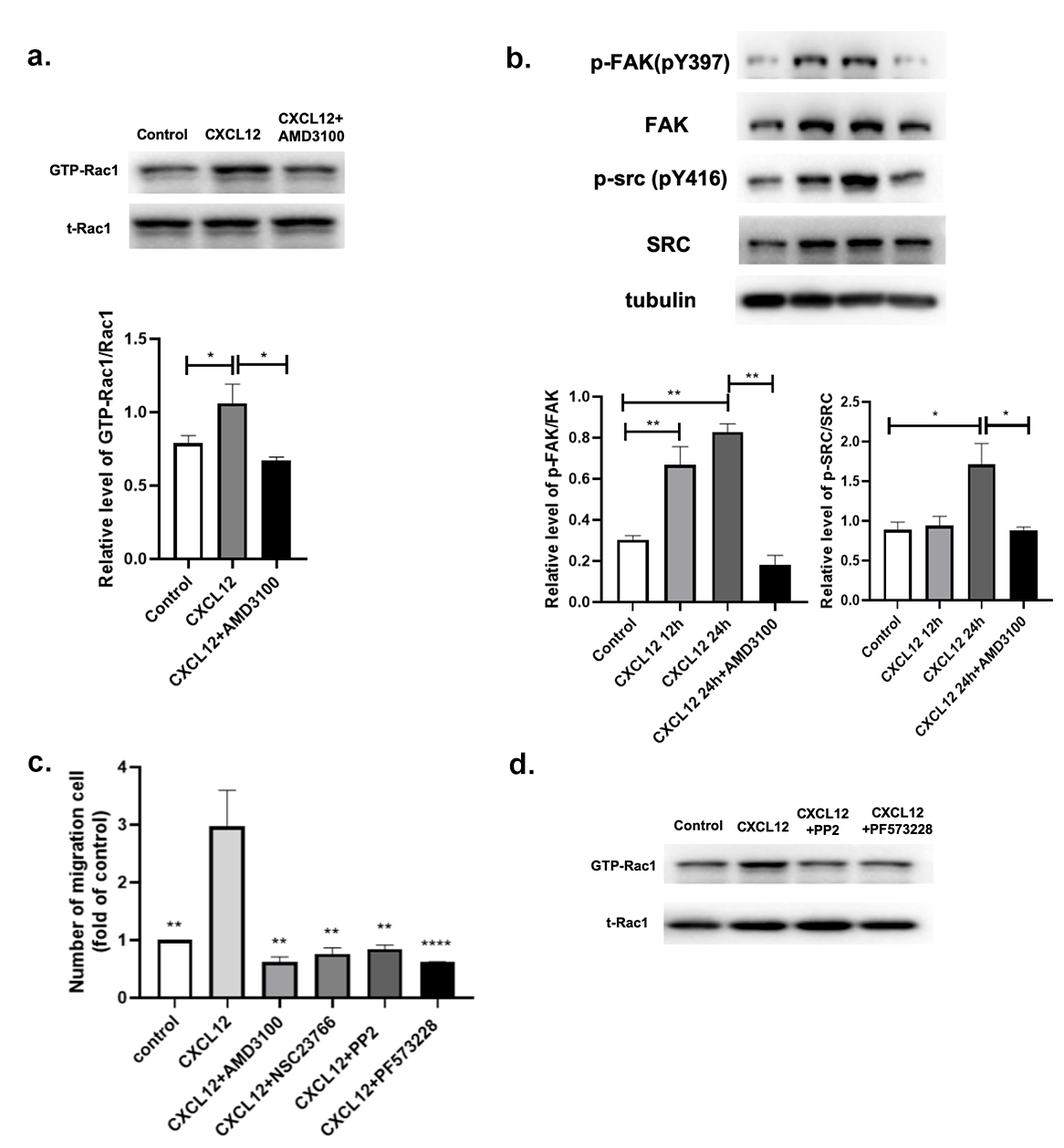
Figure S6. Verification of FAK/Src/Rac-1 signaling induced by CXCL12 in primary microglia.** (a) The expression levels of GTP-Rac1 and total Rac1 were detected by western blotting after 24 h of stimulation by CXCL12 with or without AMD3100. (b) Western blot showed the expression levels of phospho-FAK (pY397), FAK, phospho-Src (pY416) and Src after stimulation with CXCL12. (c) Migration of primary microglia towards CXCL12 with or without inhibitors was measured by the Transwell assay. (d) Expression of GTP-Rac1 and total Rac1 were detected by western blotting after 24 h of stimulation by CXCL12 with or without PP2 or PF573228. Data were shown as the mean ± SEM from three independent experiments. ****p < 0.0001. ***p < 0.001. **p < 0.01. *p < 0.05. BSA served as a control.
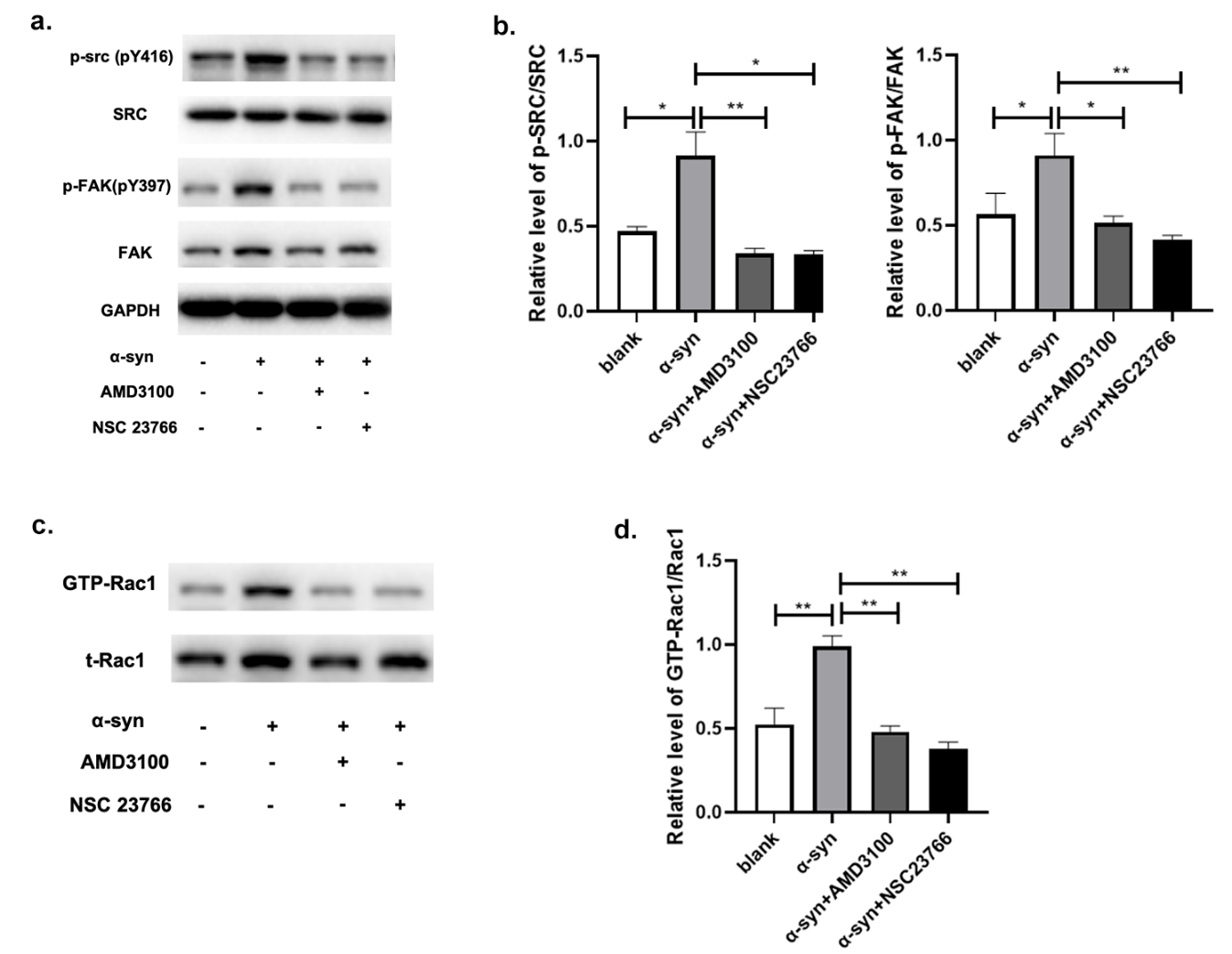


**Figure S7. Verification of FAK/Src/Rac-1 signaling induced by α-synuclein in primary microglia.** (a)(b) Western blot analysis was used to assess the expression of phospho-FAK (pY397), FAK, phospho-Src (pY416), Src, GTP-Rac1 and total Rac1 after stimulation (α-synuclein with or without AMD3100 and NSC23766).

**
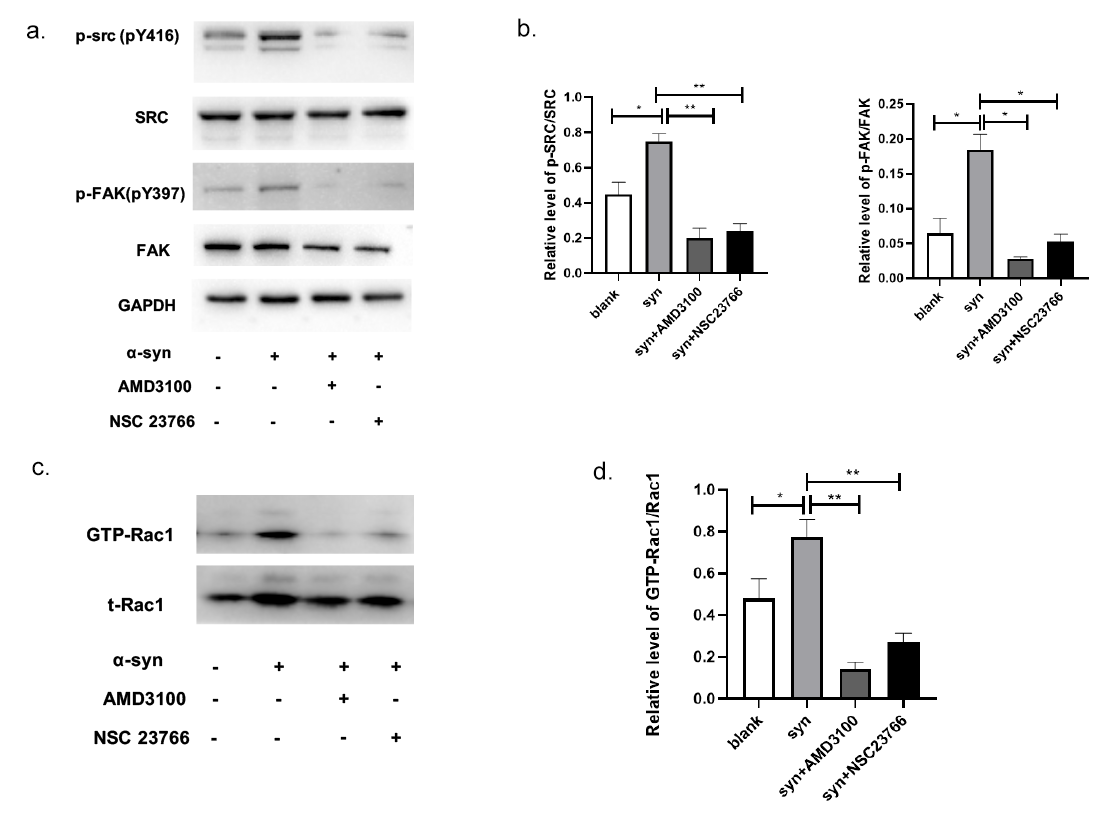
**

**Figure S8. Verification of FAK/Src/Rac-1 signaling induced by α-synuclein in RAW 264.7 cells.** (a)(b) Western blot analysis was used to assess the expression of phospho-FAK (pY397), FAK, phospho-Src (pY416), Src, GTP-Rac1 and total Rac1 after stimulation (α-synuclein with or without AMD3100 and NSC23766).
